# Trajectories of physical function and quality of life in people with osteoarthritis: results from a 10-year population-based cohort

**DOI:** 10.1186/s12889-023-16167-9

**Published:** 2023-07-21

**Authors:** Daniela Costa, David G. Lopes, Eduardo B. Cruz, Ana R. Henriques, Jaime Branco, Helena Canhão, Ana M. Rodrigues

**Affiliations:** 1 NOVA National School of Public Health, Public Health Research Centre, Universidade NOVA de Lisboa, Avenida Padre Cruz, 1600-560 Lisbon, Portugal; 2 Comprehensive Health Research Centre (CHRC), NOVA Medical School, Universidade Nova de Lisboa, Lisbon, Portugal; 3 EpiDoC Unit, NOVA Medical School, Universidade NOVA de Lisboa, Lisbon, Portugal; 4 Physiotherapy Department, School of Health, Polytechnic Institute of Setúbal, Setúbal, Portugal; 5 Centro Hospitalar Lisboa Ocidental (CHLO-E.P.E.), Serviço de Reumatologia Do Hospital Egas Moniz, Lisbon, Portugal; 6 Rheumatology Unit, Hospital Dos Lusíadas, Lisbon, Portugal

**Keywords:** Osteoarthritis, Trajectories, Health-Related Quality of Life, Physical Function, Prognosis

## Abstract

**Objective:**

To identify long-term trajectories of physical function and health-related quality of life (HRQoL) among people with hip and/or knee osteoarthritis (HKOA) and the sociodemographic, lifestyle, and clinical factors associated with different trajectories.

**Methods:**

Participants with HKOA from the EpiDoC study, a 10-year follow-up (2011–2021) population-based cohort, were considered. Sociodemographic, lifestyle, and clinical variables were collected at baseline in a structured interview and clinical appointment. Physical function and HRQoL were evaluated with the Health Assessment Questionnaire (HAQ) and EuroQoL, respectively, at baseline and the three follow-ups. Group-based trajectory modeling identified physical function and HRQoL trajectories. Multinomial logistic regression analyzed the associations between the covariates of interest and trajectory assignment (*p* < 0.05).

**Results:**

We included 983 participants with HKOA. We identified three trajectories for each outcome: “consistently low disability” (32.0%), “slightly worsening moderate disability” (47.0%), and “consistently high disability” (21.0%) for physical function; “consistently high HRQoL” (18.3%), “consistently moderate HRQoL” (48.4%) and “consistently low HRQoL” (33.4%) for HRQoL. Age ≥ 75 years, female sex, multimorbidity, and high baseline clinical severity were associated with higher risk of assignment to poorer physical function and HRQoL trajectories. Participants with high education level and with regular physical activity had a lower risk of assignment to a poor trajectory. Unmanageable pain levels increased the risk of assignment to the “consistently moderate HRQoL” trajectory.

**Conclusion:**

Although the trajectories of physical function and HRQoL remained stable over 10 years, approximately 70% of people with HKOA maintained moderate or low physical function and HRQoL over this period. Modifiable risk factors like physical activity, multimorbidity and clinical severity were associated with poorer physical function and HRQoL trajectories. These risk factors may be considered in tailored healthcare interventions.

**Supplementary Information:**

The online version contains supplementary material available at 10.1186/s12889-023-16167-9.

## Introduction

Osteoarthritis (OA) is the most common joint disease, affecting 519 million people worldwide in 2019 [1]. The hip and knee are the joints most affected by OA, responsible for 9.6 million years lived with disability [2]. Hip and/or knee OA (HKOA) comes at the high cost of up to 1%–2.5% of the gross domestic product of high-income countries due to the high utilization of healthcare services, mostly for patients requiring total joint replacement surgery. The high socioeconomic burden of HKOA is also due to the absenteeism, early retirement, and loss of productivity caused by this condition [3].

People with HKOA experience acute and chronic pain and limitations on physical function as well as progressive negative consequences for their mental health, health-related quality of life (HRQoL), and participation in social, leisure, and occupational activities [4]. OA is a long-course, fluctuating, and complex disease with varying clinical characteristics and heterogenous progression [5]. This multidimensionality challenges the prediction of the evolution of clinical symptoms and the long-term impact of the disease in physical function and HRQoL [6], and disease progression and phenotypes have been suggested as top priorities issues for OA research [7].

Few studies have analyzed the long-term trajectories of physical function and HRQoL in people with HKOA. The systematic review by Wieczorek et al. (2020) has suggested that pain and physical function trajectories are stable over time, but some people might improve their symptoms (6). HRQoL has been a less studied outcome, and to our knowledge, only two studies from the Osteoarthritis Iniciative analyzed the trajectory of HRQoL in the long term, in people with knee OA, with similar results [8]. Studies have reported follow-ups from 12 weeks to 8 years, and a high heterogeneity was noted in the number of trajectories found, the outcomes analyzed, and statistical methods used [6]. As a chronic health condition, longer follow-ups are needed to fully understand the progression of HKOA. Understanding the different trajectories of physical function and HRQoL in people with HKOA in the long term, and factors that may predict HKOA trajectories may allow clinicians to individualize interventions according to clinical progression [8]. Stratifying patients by their risk of high levels of disability and worsening quality of life and delivering targeted treatment interventions has become a key focus for OA research [7].

This study aimed to identify longitudinal trajectories of physical function and HRQoL over 10 years and identify the sociodemographic, lifestyle, and clinical variables associated with different trajectories. Secondarily, this study aimed to describe the patterns of specific dimensions of physical function and HRQoL in the 10-year period.

## Methods

### Data source

This nationwide longitudinal study in Portugal analyzed data from the Epidemiology of Chronic Diseases (EpiDoC) cohort (2011–2021), which was comprised of randomly selected Portuguese adults (≥ 18 years old) living in private households, as previously described in the literature [9]. The EpiDoC cohort had four waves: EpiDoC 1 (*N* = 10,661) collected baseline data from September 2011 to December 2013; EpiDoC 2 (*N* = 7,591) started in March 2013 and ended in July 2015; EpiDoC 3 (*N* = 5,653) started in September 2015 and ended in July 2016; and the most recent wave, EpiDoC 4 (*N* = 3,757), occurred from March to August 2021. The baseline evaluation (EpiDoC 1) aimed to estimate the prevalence of 12 rheumatic and musculoskeletal diseases (RMDs) in Portugal, including HKOA. EpiDoC 1 was performed in two phases. In the first phase, trained research assistants conducted face-to-face interviews with a structured questionnaire to collect data on socioeconomic status, chronic non-communicable diseases, HRQoL, and healthcare resource consumption and screen for RMDs. The second phase integrated a clinical appointment for all participants who screened positive for RMDs and 20% of those with negative RMD screenings who agreed to participate. Each appointment consisted of a structured evaluation with a rheumatologist — including laboratory and imaging exams, if needed — to validate the RMD diagnosis and evaluate the patient’s disease-related information [9]. In this phase, data from 3877 participants were collected. The participants who attended the clinical appointments did not differ from those who did not except for age group, gender and residence region, as previously described [9].

**Fig. 1:**
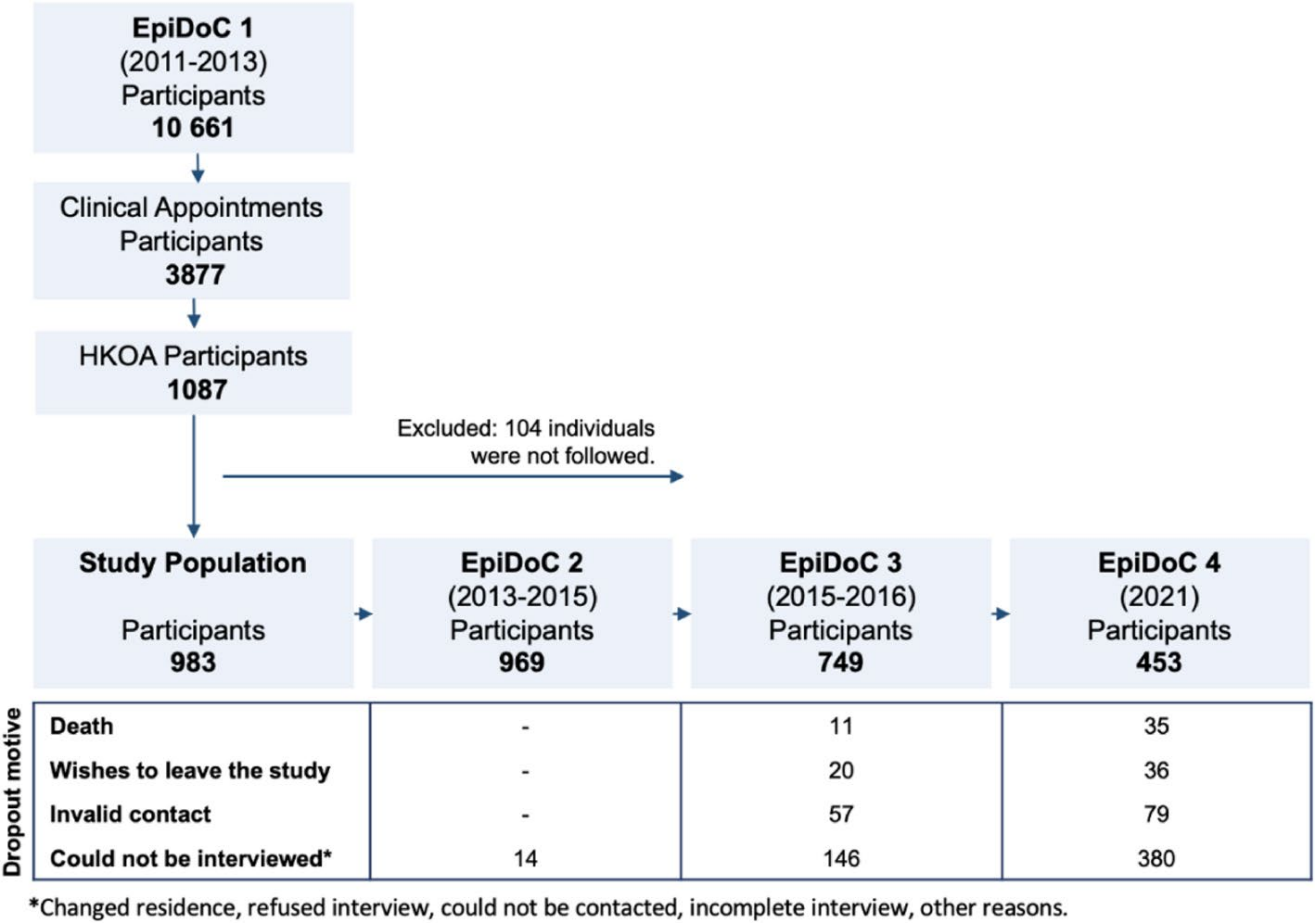
Flowchart of study design for participants with hip/knee osteoarthritis

In each follow-up wave, trained research assistants performed follow-up evaluations as an interview over the phone. These interviews were guided by a core questionnaire to collect data on socioeconomic status, new diagnoses of chronic non-communicable diseases, HRQoL, physical function, and healthcare resource consumption to gather longitudinal data. Each wave also had specific questions on lifestyles and health-related issues to enable the collection of cross-sectional and longitudinal data [9, 10] – Fig. 1.

### Study population

This study included participants from the EpiDoC cohort with an HKOA diagnosis validated by a rheumatologist during EpiDoC 1 according to the American College of Rheumatology OA classification criteria for hip [11] and knee OA [12]. Additionally, participants were only included if they had participated in at least one follow-up wave. Therefore, we excluded participants with HKOA diagnosis who did not participate in any of the follow-up waves. Of the 983 participants included in EpiDoC 1, 98.6% (*n* = 969), participated in EpiDoC 2, 76.2% (*n* = 749) participated in EpiDoC 3 and 46.1% (*n* = 453) participated in EpiDoC 4 – Fig. 1.

### Attrition analysis

Slight differences were noted between participants who dropped out, and included participants. Participants who dropped out were older, with lower HRQoL and physical function, higher clinical severity and encompassed a higher proportion of people with multimorbidity, without partner and lower education, when compared to the ones that were included – Additional File: Table S 2. On average, included participants provided 3.1 ± 0.8 timepoint measures of EQ5D-3L and HAQ scores. Total sample average years of EpiDoC 4 (2021) to baseline (EpiDoC 1) were 8.38 ± 0.61 years – Additional File: Table S 3.

### Outcome definition and measurements

#### Physical function

Physical function was measured with the Health Assessment Questionnaire (HAQ), that is the most widely used questionnaire to assess functional status in patients with arthritis. Although not specific for people with HKOA, it was previously tested in this population, showing good psychometric properties [13]. HAQ was used in the first phase of EpiDoC 1, as a baseline and by a phone interview during the three follow-up assessments. This instrument evaluates functional impairments in 20 activities of daily living classified into eight dimensions: dressing and grooming, arising, eating, walking, hygiene, reach, grip, and common daily activities (e.g., shopping, entering and exiting a car, and doing chores). Each activity was scored from 0 to 3 according to the individual’s difficulty in performing it: 0, “Without any difficulty”; 1, “With some difficulty”; 2, “With much difficulty”; and 3, “Unable to do”. The worst score in each of the eight dimensions was then summed and divided by the number of dimensions. The total possible scores lie between zero, indicating no functional impairment/disability, and 3, indicating complete impairment/disability [13].

#### Health-related quality of life

HRQoL was measured with the Portuguese version of EuroQoL, with a 5-dimension and 3-level (EQ-5D-3L) descriptive system. The assessment took place in the first phase of EpiDoC 1 at baseline and during the three follow-up assessments, by phone interview. This instrument describes health status in five dimensions: mobility, self-care, usual activities, pain/discomfort, and anxiety/depression. Each dimension is scored within three levels (without problems, some problems, extreme problems). Participants were asked to mark the option that would best describe their experience on the day of the interview. This descriptive system was then computed to a single summary index score using a valuation algorithm based on interviews from the general Portuguese population (weights from a representative national sample). In this study, the EQ-5D-3L index score ranged from 1 to -0.59, where 1 represents “full health”, 0 represent health states equivalent to “death” and scores less than 0 represent health states that are considered worse than being dead [14].

#### Covariates of interest

Sociodemographic, lifestyle, and clinical variables were collected during the baseline assessment. Given the scarcity of data in some categories, and to ensure optimal interpretation of the data, several variables were subjected to categorical transformation.

In this study, we considered age class, sex, and geographic location — according to NUTS II territorial units (Lisbon, North, Centre, Algarve, Alentejo, Madeira, and Azores)—as sociodemographic variables. The age classes were < 55 years old, 55–64 years old, 65–74 years old, and ≥ 75 years old. In the analysis of geographic locations, Madeira and Azores were merged to form one “Islands” region. Marital status was categorized as “with partner” (married or consensual union) and “no partner” (single, widowed, or divorced). Education level was categorized according to the years of education completed: “ < 4 years” (less than primary education), “4–9 years” (primary or secondary education), and “ ≥ 10 years” (secondary or higher education). Body mass index (BMI) was categorized as “underweight” (≤ 18.49 kg/m^2^), “healthy weight” (≥ 18.5 and ≤ 24.99 kg/m^2^), “overweight” (≥ 25 and ≤ 29.99 kg/m^2^), and “obese” (≥ 30 kg/m^2^) according to self-reported height and weight. Lifestyle variables were collected as well, including smoking habits (“never,” “in the past,” and “occasionally or daily”), and whether individuals participated in regular physical activity/sports (“yes”, “no”, and “doesn’t know/doesn’t answer”). Alcohol intake variable was categorized as “occasionally or daily” or “never”, characterizing participants that consume and participants that do not consume alcohol, respectively.

Multimorbidity was defined as having two or more self-reported chronic non-communicable diseases from the following list noted in the baseline assessment: high blood pressure, high cholesterol, cardiac disease, diabetes mellitus, chronic lung disease, problems in the digestive tract, neurological disease, mental disease, allergies, cancer, and hyperuricemia [15].

Clinical severity was evaluated at baseline with the Portuguese versions of the Knee Injury and Osteoarthritis Outcome Score (KOOS) [16] and the Hip Disability and Osteoarthritis Outcome Score (HOOS) [17]. HOOS/KOOS are self-reported assessments that evaluate the consequences of HKOA in five dimensions: pain, other symptoms, activities of daily living, sports and leisure, and quality of life. Scores for each dimension were transformed to a 0–100 scale, with 0 representing extreme hip/knee problems and 100 representing no hip/knee problems [16, 17]. A final composite score was calculated with the mean score of each dimension, as previously recommended [18]. For this study, and to facilitate the interpretation of this measure, the final score is reported as the inverted normalized mean score (0–100), with higher values corresponding to higher clinical severity, as previously documented [19]. Pain intensity was measured as the mean pain intensity in the previous week with the 11-point Numeric Pain Rating Scale (NPRS) at baseline. Zelman et al. (2003), using question 5 of the Brief Pain Inventory scale to determine the average pain in the previous week on an 11-point NPRS, found 5 points to be the optimal cut-off point to consider a pain day manageable in OA (F [7, 9] = 7.08, *p* < 0.001) [20]. Therefore, we divided the population into two subgroups: manageable pain levels (< 5 points) and unmanageable pain levels (≥ 5 points).

These covariates are clinically important variables that, according to literature, can potentially influence health outcomes in people with HKOA [21]. Independent from HKOA, age and sex can also generally influence physical function and quality of life (outcomes). Age and sex are also related with the presence of comorbidities, socio-economic status and other health variables (independent variables) [22]. Therefore, these two variables were included as confounders in the analysis. In case of more than one joint affected, we considered the joint with the worst score in KOOS/HOOS and in NPRS.

### Data analysis

We first performed descriptive analysis of the HAQ and EQ-5D-3L dimensions for each of the follow-ups in the 10-year period. The proportions of participants that reported “some difficulty” and “with much difficulty” in each HAQ dimension and “some problems” or “extreme problems” in each EQ-5D-3L dimension were computed and plotted, separately, for better interpretation – Additional File: Figure S 1 and S 2.

We used a group-based trajectory modeling analysis to identify different trajectories of physical function and HRQoL over the 10-year period. For this, we considered only HKOA patients who participated in both the baseline assessment and at least one of the cohort follow-ups. Group-based trajectory modeling uses finite mixtures of probability distributions based on maximum likelihood estimation to identify clusters of individuals with similar trajectories.

Posterior probabilities were estimated to quantify the likelihood of an individual belonging to a specific trajectory, and participants were placed into their respective trajectories with the highest posterior probability. We considered and tested models with three, four and five trajectories – Additional File: Table S 5, Figure S 4. The final model was chosen based on a combination of several criteria, as recommended [23]. We considered the log of the Bayes factor approximation, by comparing changes in the Bayesian Information Criteria (BIC) between models, in which lower BIC values indicate a better model fit [24]. Odds of correct classification and average posterior probabilities were also considered. Nagin (2010) recommended that average posterior probabilities should be ≥ 0.7, as the optimal cut-off [23]. To ensure further statistical analysis we considered that each trajectory should include at least *n* = 100 participants, approximately 10% of the sample size. With this information, we finally considered the model with a meaningful pragmatic interpretability, that better describes different patterns of change from a clinical perspective [23].

A censored normal distribution specification was considered for both outcomes (EQ-5D-3L and HAQ scores). In this approach, negative EQ-5D-3L scores were recoded and attributed a value of 0 for compatibility because, theoretically, 0 and values below 0 represent a low HRQoL state (n_recoded_ = 63). Therefore, in this analysis, the EQ-5D-3L index scores ranged from 0 to 1.

Descriptive analysis was performed for the sociodemographic, clinical, and lifestyle characteristics of the study population and each of the trajectory subgroups using absolute and relative frequencies for categorical variables and the mean and standard deviation for continuous variables. The same analyses were conducted separately for the participants included in each wave (Additional File: Table S 1). Independence hypotheses were tested to compare the different trajectory subgroups according to their sociodemographic, clinical, and lifestyle characteristics using non-parametric tests: Chi-squared for categorical variables and Kruskal–Wallis for continuous variables. Independency tests were also used to compare included and drop-out participants in each wave (Additional File: Table S 2).

Finally, we used a 2-step multinomial logistic regression model to assess the associations between the baseline variables, namely the sociodemographic, clinical and lifestyle variables, and trajectory groups assignment. In the first step, we conducted a univariate analysis, considering a significance level of 0.25 to avoid early exclusion of potentially important variables. Additionally, multicollinearity was checked with all independent variables included in the multinomial model, showing variance inflation factor (VIF) values below 3 [25]. Then, with a forward conditional method, we sequentially included the statistically significant variables and compared the models through likelihood ratio tests based on the Akaike Information Criterion until the final models were reached. The relative risk ratio (RRR) was estimated for each variable with a 95% confidence interval (95% CI).

The models’ postestimation was evaluated through a generalized Hosmer–Lemeshow goodness-of-fit test [26] under the null hypothesis that the model fit the data correctly, i.e., the observed and expected frequencies did not differ significantly.

The missing data for covariates was below 10% thus, no imputation methods were used. Participants with missing data on the multinomial logistic regression analysis were automatically excluded in this procedure, constituting a complete case analysis. The adjustment of sex and age was forced in the models. Due to scarcity of data, the normal and underweight BMI categories were merged into one (< 25.00 kg/m^2^).

All analyses were performed with STATA v16.1 considering a level of significance of 0.05. Trajectory analysis was carried out using the *traj* plugin [23].

## Results

Of the 983 participants with HKOA from the EpiDoC cohort, 96 were diagnosed with hip OA, 803 with knee OA, and 84 with hip and knee OA.

### Patterns of physical function and HRQoL dimensions over time

Considering physical function, EpiDoC 2 was the wave with the highest percentage of people that reported “some” or “much difficulty” in all dimensions of HAQ. Namely, in “reach” this proportion was 78.12% (*n* = 739), in “walking” 70.59% (*n* = 672) and in arising 68.73% (*n* = 655). These were also the dimensions with the highest proportion of people who experienced some or much difficulty in all four waves. “Walking” was the dimension with the largest increase in the proportion of people who reported some or much difficulty between EpiDoC 1 (*n* = 505, 51.37%) and EpiDoC 4 (*n* = 262, 64.37%) – Additional File: figure S 1, Table S 4.

Overall, the patterns in the EQ-5D-3L dimensions over the 10-year period were similar to those in the HAQ dimensions. Pain and mobility were the dimensions for which the largest proportions of people with HKOA reported some or extreme problems: 74.87% (*n* = 715) and 70.87% (*n* = 674) in EpiDoC 2, respectively. Self-care dimension had the greatest increase in the proportion of people reporting some or extreme problems over the 10-year period (EpiDoC 1: *n* = 194, 19.73%; EpiDoC 4: *n* = 125, 30.56%)—Additional File: figure S 2, Table S 4.

### Physical function and HRQoL trajectories

**Fig. 2:**
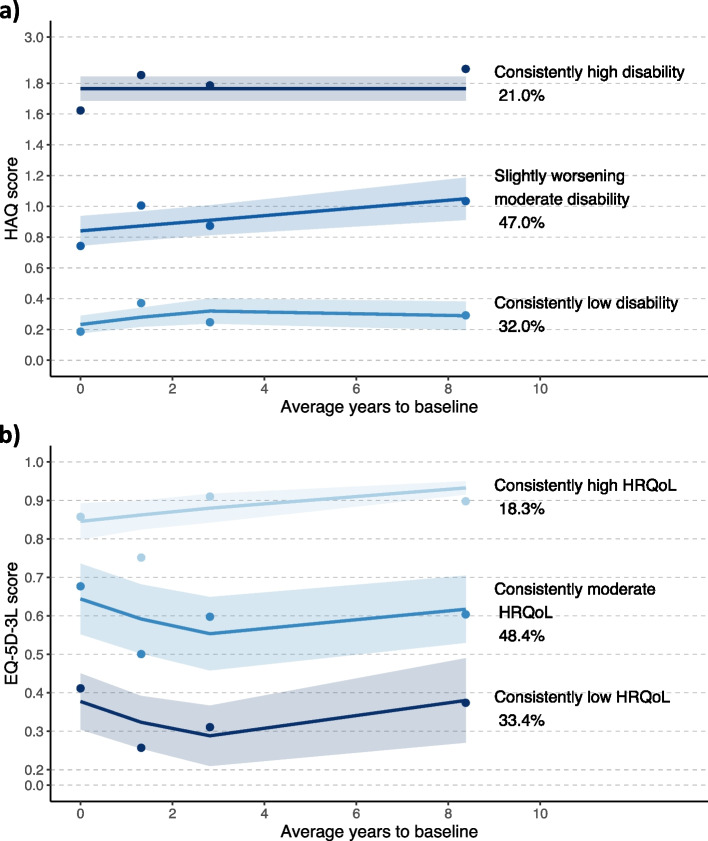
Estimated **a**) physical function and **b**) HRQoL trajectories for people with hip and/or knee osteoarthritis, and proportion of individuals in each group. The horizontal axis includes the follow-up time and the vertical axis represents the final score of a) HAQ (min–max: 0–3), in the case of physical function, and b) EQ-5D-3L (min–max: 0–1), in case of HRQoL trajectories. Shapes represent observed mean estimates and lines represent the predicted mean estimates. Y axis scale was partially collapsed and some values were omitted for better visualization

Based on the BIC values and clinical interpretation of trajectories, a model with three trajectory groups was achieved for both physical function and HRQoL (Additional File: table S 5) with an average posterior probability of group membership greater than 0.7 (Additional File: Table S 6). Trajectories of physical function in the 10-year follow-up were identified as: 1) “consistently high disability” (*n* = 204, 21.0%); 2) “slightly worsening moderate disability” (*n* = 472, 47.0%); and 3) “consistently low disability” (*n* = 307, 32.0%) – Fig. 2 a). For HRQoL, the three trajectories were defined as: 1) “consistently low HRQoL” (*n* = 317, 33.4%); 2) “consistently moderate HRQoL” (*n* = 501, 48.4%); 3) “consistently high HRQoL” (*n* = 165, 28.3%), where participants consistently reported low, moderate and high HRQoL during the follow-up, respectively – Fig. 2b).

### Baseline characteristics of participants with HKOA according to trajectory assignment

The HKOA participants (*n* = 983) had a mean age of 62.2 ± 11.2 years old, 71.3% (*n* = 701) were female, 81.7% (*n* = 751) were overweight or obese, multimorbidity was present in 70.8% (*n *= 634) of the participants, and only 21.6% (*n* = 212) reported regular physical activity (Table 1).

**Table 1:** Baseline sociodemographic, anthropometric, lifestyle and clinical characteristics for the study population and Physical Function trajectory groups

	**Total Sample** *n* = 983	**Physical Function (HAQ score) Trajectories**			
		Consistently high disability *n* = 204	Slightly worsening disability *n* = 472	Consistently low disability *n* = 307	*p*^*a*^
Sociodemographic					
Age (years)					
Mean (SD)	65.2 (11.2)	70.9 (9.4)	65.4 (10.6)	61.2 (11.5)	< 0.001
< 55 years old, n (%)	170 (17.3%)	13 (6.4%)	79 (16.7%)	78 (25.4%)	< 0.001
55–64 years old, n (%)	265 (27.0%)	34 (16.7%)	128 (27.1%)	103 (33.6%)	
65–74 years old, n (%)	340 (34.6%)	78 (38.2%)	168 (35.6%)	94 (30.6%)	
≥ 75 years old, n (%)	208 (21.2%)	79 (38.7%)	97 (20.6%)	32 (10.4%)	
Female sex, n(%)	701 (71.3%)	176 (86.3%)	359 (76.1%)	166 (54.1%)	< 0.001
Region (NUTS II), n (%)					
North	267 (27.2%)	65 (31.9%)	125 (26.5%)	77 (25.1%)	0.032
Center	238 (24.2%)	47 (23.0%)	118 (25.0%)	73 (23.8%)	
Lisbon	165 (26.8%)	31 (15.2%)	66 (14.0%)	68 (22.1%)	
Alentejo	58 (5.9%)	15 (7.4%)	28 (5.9%)	15 (4.9%)	
Algarve	20 (2.0%)	7 (3.4%)	6 (1.3%)	7 (2.3%)	
Islands	235 (23.9%)	39 (19.1%)	129 (27.3%)	67 (21.8%)	
Marital status, partner n (%)	641 (65.2%)	112 (54.9%)	316 (67.0%)	213 (69.4%)	0.002
Educational level, n (%)					
< 4 years	230 (23.4%)	92 (45.1%)	107 (22.7%)	31 (10.1%)	< 0.001
4–9 years	630 (64.1%)	106 (52.0%)	314 (66.5%)	210 (68.4%)	
≥ 10 years	123 (12.5%)	6 (2.9%)	51 (10.8%)	66 (21.5%)	
Anthropometric					
BMI (kg/m^2^), n (%)					
Underweight/Normal weight	168 (18.3%)	32 (18.1%)	71 (16.0%)	65 (21.9%)	< 0.001
Overweight	376 (40.9%)	62 (35.0%)	169 (38.0%)	145 (48.8%)	
Obese	375 (40.8%)	83 (46.9%)	205 (46.1%)	87 (29.3%)	
Lifestyle					
Smoking habits n (%)					
Never	732 (74.5%)	173 (84.8%)	371 (78.6%)	188 (61.2%)	< 0.001
In the past	180 (18.3%)	20 (9.8%)	71 (15.0%)	89 (29.0%)	
Daily/Occasionally	71 (7.2%)	11 (5.4%)	30 (6.4%)	30 (9.8%)	
Alcohol consumption n (%)					
Never	497 (50.6%)	126 (61.8%)	251 (53.3%)	120 (39.1%)	< 0.001
Occasionally/Daily	485 (49.4%)	78 (38.2%)	220 (46.7%)	187 (60.9%)	
Regular physical activity n (%)	212 (21.6%)	23 (11.3%)	91 (19.3%)	98 (32.0%)	< 0.001
Clinical					
Multimorbidity n (%) yes	634 (70.8%)	158 (91.9%)	313 (73.0%)	163 (55.4%)	< 0.001
Unmanageable pain levels (≥ 5 NPRS), n (%)	691 (73.8%)	164 (85.0%)	355 (78.2%)	172 (59.5%)	< 0.001
Clinical severity mean(SD) (Inverted HOOS/KOOS)	46.1 (18.8)	59.4 (15.1)	48.8 (16.0)	33.1 (17.1)	< 0.001
Physical function (HAQ score) – Mean (SD)	0.8 (0.7)	1.7 (0.5)	0.8 (0.5)	0.2 (0.2)	< 0.001
HRQoL (EQ5D score) – Mean (SD)	0.6 (0.3)	0.4 (0.2)	0.6 (0.2)	0.8 (0.2)	< 0.001

^a^*p*-values for non-parametric independency tests (Chi-squared for categorical variables, Kruskal–Wallis for continuous variables)

*BMI* Body mass index, *NPRS* Numeric Pain Rating Scale, *HRQoL* Health-Related Quality of Life

Sample size is not constant due to missing values in some variables at baseline: BMI (*n* = 64); Alcohol consumption (*n* = 1); Regular physical activity (*n* = 1); Multimorbidity (*n* = 88); Clinical severity (*n* = 80); Unmanageable pain levels (*n* = 47); HRQoL, EQ-5D-3L (*n* = 12)

For physical function, the “consistently high disability” trajectory group had the highest mean age (70.9 ± 9.4 years, *p* < 0.001), the largest proportion of female participants (*n* = 176, 86.3%, *p* < 0.001), with low education level (< 4 years of education; *n* = 92, 45.1%, *p* < 0.001), obesity (*n* = 83, 46.9%, *p* < 0.001), no regular physical activity (*n* = 23, 11.3%, *p* < 0.001), multimorbidity (*n* = 155, 89.1%, *p* < 0.001), unmanageable pain levels (*n* = 165, 85.0%, *p* < 0.001) and the highest clinical severity (inverted HOOS/KOOS5: 59.4 ± 15.1, *p* < 0.001)—Table 1.

Similarly, the “consistently low HRQoL” trajectory group had the highest mean age (68.4 ± 10.1 years old, *p* < 0.001), the largest proportions of female participants (*n* = 264, 83.3%, *p* < 0.001) and participants with the lowest education level (< 4 years of education: *n* = 125, 39.4%, *p* < 0.001), with obesity (*n* = 150, 53.0%, *p* < 0.001), no regular physical activity (*n* = 37, 11.7%, *p* < 0.001), multimorbidity (*n* = 230, 83.3%, *p* < 0.001), unmanageable pain levels (*n* = 256, 84.2%, *p* < 0.001) and the highest clinical severity (inverted HOOS/KOOS: 57.0 ± 15.6, *p* < 0.001) – Table 2.

**Table 2:** Baseline sociodemographic, anthropometric, lifestyle and clinical characteristics for participants assigned to the HRQoL trajectory groups

		**HRQoL (EQ5D score) Trajectories**			*p*^*a*^
	Total Sample *n* = 983	Consistently low HRQoL *n* = 317	Consistently moderate HRQoL *n* = 501	Consistently high HRQoL *n* = 165	
Sociodemographic					
Age (years)					
Mean (SD)	65.2 (11.2)	68.4 (10.1)	64.7 (10.9)	60.5 (12.0)	< 0.001
< 55 years old, n (%)	170 (17.3%)	35 (11.0%)	92 (18.4%)	43 (26.1%)	< 0.001
55–64 years old, n (%)	265 (27.0%)	71 (22.4%)	141 (28.1%)	53 (32.1%)	
65–74 years old, n (%)	340 (34.6%)	114 (36.0%)	172 (34.3%)	54 (32.7%)	
≥ 75 years old, n (%)	208 (21.2%)	97 (30.6%)	96 (10.2%)	15 (9.1%)	
Female Sex, n(%)	701 (71.3%)	264 (83.3%)	350 (69.9%)	87 (52.7%)	< 0.001
Region (NUTS II), n (%)					
North	267 (27.2%)	95 (30.0%)	127 (25.3%)	45 (27.3%)	0.920
Center	238 (24.2%)	75 (23.7%)	127 (25.3%)	36 (21.8%)	
Lisbon	165 (26.8%)	47 (14.8%)	85 (17.0%)	33 (20.0%)	
Alentejo	58 (5.9%)	18 (5.7%)	31 (6.2%)	9 (5.5%)	
Algarve	20 (2.0%)	7 (2.2%)	9 (1.8%)	4 (2.4%)	
Islands	235 (23.9%)	75 (23.7%)	122 (24.4%)	38 (23.0%)	
Marital status, partner, n (%)	641 (65.2%)	190 (59.9%)	323 (64.5%)	128 (77.6%)	0.001
Educational level, n (%)					
< 4 years	230 (23.4%)	125 (39.4%)	88 (17.6%)	17 (10.3%)	< 0.001
4–9 years	630 (64.1%)	179 (56.5%)	344 (68.7%)	107 (64.8%)	
≥ 10 years	123 (12.5%)	13 (4.1%)	69 (13.8%)	41 (24.9%)	
Anthropometric					
BMI (kg/m^2^), n (%)					
Underweight/Normal weight	168 (18.3%)	39 (13.8%)	98 (20.6%)	31 (19.5%)	< 0.001
Overweight	376 (40.9%)	94 (33.2%)	199 (41.7%)	83 (52.2%)	
Obese	375 (40.8%)	150 (53.0%)	180 (37.7%)	45 (28.3%)	
Lifestyle					
Smoking habits n (%)					
Never	732 (74.5%)	257 (81.1%)	370 (73.8%)	105 (63.6%)	< 0.001
In the past	180 (18.3%)	41 (12.9%)	87 (17.4%)	52 (31.5%)	
Daily/Occasionally	71 (7.2%)	19 (6.0%)	44 (8.8%)	8 (4.9%)	
Alcohol consumption n (%)					
Never	497 (50.6%)	180 (56.8%)	260 (52.0%)	57 (34.6%)	< 0.001
Occasionally/Daily	485 (49.4%)	137 (43.2%)	240 (48.0%)	108 (65.4%)	
Regular physical activity n (%)	212 (21.6%)	37 (11.7%)	117 (23.4%)	58 (35.2%)	< 0.001
Clinical					
Multimorbidity n (%)	634 (70.8%)	242 (88.3%)	305 (65.9%)	87 (55.1%)	< 0.001
Unmanageable pain levels (≥ 5 NPRS), n (%)	691 (73.8%)	256 (84.2%)	357 (75.0%)	78 (50.0%)	< 0.001
Clinical severity, Mean (SD) (Inverted HOOS/KOOS)	46.1 (18.8)	57.0 (15.6)	44.1 (17.1)	31.4 (17.3)	< 0.001
Physical function (HAQ score) – Mean (SD)	0.8 (0.7)	1.3 (0.6)	0.6 (0.5)	0.3 (0.4)	< 0.001
HRQoL (EQ5D score) – Mean (SD)	0.6 (0.3)	0.4 (0.2)	0.70 (0.2)	0.9 (0.2)	< 0.001

^a^*p*-values for non-parametric independency tests (Chi-squared for categorical variables, Kruskal–Wallis for continuous variables)

*BMI* Body mass index, *NPRS* Numeric Pain Rating Scale, *HRQoL* Health-Related Quality of Life

Sample size is not constant due to missing values in some variables at baseline: BMI (*n* = 64); Alcohol consumption (*n* = 1); Regular physical activity (*n* = 1); Multimorbidity (*n* = 88); Clinical severity (*n* = 80); Unmanageable pain levels (*n* = 47); HRQoL, EQ-5D-3L (*n* = 12)

For both outcome measures, participants showed poorer mean scores in the poorer trajectory groups—i.e., “consistently high disability” (HAQ: 1.7 ± 0.5; *p* < 0.001) and “consistently low” HRQoL (EQ-5D-3L: 0.4 ± 0.2; *p* < 0.001) – Tables 1 and 2.

### Baseline factors associated with physical function and HRQoL trajectory groups

Univariate logistic regression analysis is presented in the Additional File: tables S 7 and S 8, for HRQoL and physical function, respectively.

In the final multinomial logistic regression model for the physical function trajectory groups, using “consistently low disability” trajectory as reference, female participants (RRR = 2.90; 95% CI: 1.97, 4.28) and people with multimorbidity (RRR = 1.66; 95% CI: 1.13, 2.42) had a significantly higher risk of a “slightly worsening moderate disability”. Female participants (RRR: 5.56; 95% CI: 2.99, 10.34), adults aged 75 years and over (RRR = 3.93; 95% CI: 1.48, 10.46), and people with multimorbidity (RRR = 4.99; 95% CI: 2.49, 10.00) had a higher risk of assignment to the “consistently high disability” trajectory. Baseline clinical severity increased the likelihood of being assigned to the “consistently high disability” (RRR = 1.09; 95% CI: 1.07, 1.11) and the “slightly worsening moderate disability” (RRR = 1.06; 95% CI: 1.04, 1.07) trajectories. People with a high level of education (≥ 10 years) (RRR = 0.19; 95% CI: 0.05, 0.64) and a baseline report of regular physical activity (RRR = 0.35; 95% CI: 0.17, 0.68) were less likely to be assigned in the “consistently high disability” trajectory – Table 3.

**Table 3:** Multinomial logistic regression model for the association of baseline characteristics and physical function (HAQ) trajectories

	Physical Function Trajectories (HAQ)		
	**Consistently low disability** **RRR (95% CI)**	**Slightly worsening moderate disability** **RRR (95% CI)**	**Consistently high disability** **RRR (95% CI)**
Sex			
Male (ref)	-	-	-
Female	-	2.90 (1.97–4.28)	5.56 (2.99–10.34)
Age class			
< 55 years old (ref)	-	-	-
55–64 years old	-	0.57 (0.34–0.97)	0.63 (0.25–1.59)
65–74 years old	-	0.93 (0.55–1.58)	1.41 (0.58–3.41)
≥ 75 years old	-	1.65 (0.86–3.17)	3.93 (1.48–10.46)
Educational level			
< 4 years (ref)	-	-	-
4–9 years	-	1.00 (0.57–1.75)	0.54 (0.28–1.03)
≥ 10 years	-	0.88 (0.44–1.78)	0.19 (0.05–0.64)
Regular physical activity (yes)	-	0.51 (0.34–0.78)	0.35 (0.17–0.68)
Clinical severity (0 best – 100 worst)	-	1.06 (1.04–1.07)	1.09 (1.07–1.11)
Multimorbidity (yes)	-	1.66 (1.13–2.42)	4.99 (2.49–10.00)

*RRR* Relative risk ratio, *HAQ* Health Assessment Questionnaire

Goodness-of-fit: *χ*^2^(16) = 19.2, df = 16, *p* = 0.259 Total sample: *n* = 823

For HRQoL, similar baseline variables were significantly associated with poor trajectory groups, using “Consistently high HRQoL” trajectory as reference class. Female sex (RRR = 2.11; 95% CI: 1.39, 3.22), older adults aged 75 years old and over (RRR = 2.65; 95% CI: 1.18, 5.92), and participants with unmanageable pain levels (RRR = 1.85; 95% CI: 1.16, 2.93) were significantly associated with a “consistently moderate HRQoL” trajectory. Female participants (RRR = 3.75; 95% CI: 2.16, 6.49) and participants with multimorbidity (RRR = 3.83; 95% CI: 2.13, 6.90) were associated with a “consistently low HRQoL trajectory”. A higher baseline clinical severity score was associated with a “consistently moderate” HRQoL (RRR: 1.03 95%CI: 1.02–1.05) and with a “consistently low” (RRR: 1.08, 95%CI: 1.06–1.10) HRQoL trajectory. Participants with a high level of education (≥ 10 years) (RRR = 0.29; 95% CI: 0.10, 0.82) and a baseline report of regular physical activity (RRR = 0.36; 95% CI: 0.20, 0.67) had a significantly lower risk of assignment to the “consistently low HRQoL” trajectory – Table 4.

**Table 4:** Multinomial logistic regression model for the association of baseline characteristics of people with HKOA and HRQoL (EQ-5D-3L) trajectories

	HRQoL Trajectories (EQ-5D)		
	**Consistently high** **HRQoL (Reference)**	**Consistently moderate** **HRQoL** **RRR (95% CI)**	**Consistently low** **HRQoL** **RRR (95% CI)**
Sex			
Male (ref)	-	-	-
Female	-	2.11 (1.39–3.22)	3.75 (2.16–6.49)
Age class			
< 55 years old (ref)	-	-	-
55–64 years old	-	0.94 (0.52–1.68)	0.68 (0.32–1.47)
65–74 years old	-	0.99 (0.55–1.79)	0.71 (0.33–1.52)
≥ 75 years old	-	2.65 (1.18–5.92)	2.24 (0.87–5.82)
Educational level			
< 4 years (ref)	-	-	-
4–9 years	-	1.32 (0.67–2.59)	0.62 (0.30–1.28)
≥ 10 years	-	1.04 (0.46–2.35)	0.29 (0.10–0.82)
Regular physical activity (yes)	-	0.60 (0.38–0.94)	0.36 (0.20–0.67)
Clinical severity (inverted KOOS/HOOS) (0 low – 100 high severity)	-	1.03 (1.02–1.05)	1.08 (1.06–1.10)
Unmanageable pain level (yes)	-	1.85 (1.16–2.93)	1.36 (0.74–2.48)
Multimorbidity	-	1.28 (0.84–1.96)	3.83 (2.13–6.90)

*RRR* Relative risk ratio, *HRQoL* Health-Related Quality of Life

Final model goodness-of-fit: *χ*^2^(16) = 17.9, *p* = 0.329 Total sample: *n* = 817

## Discussion

This study identified three different long-term trajectories of physical function (HAQ) and HRQoL (EQ-5D-3L) in people with HKOA, that remained stable over the 10-years follow-up time. Our results reveal that 68% and 81.8% of the participants live with trajectories of moderate or low physical function and HRQoL, respectively.

The trajectories found in this study are similar to previous literature published in this field. Regarding physical function, in a recent systematic review, the authors concluded that pain trajectories, as well as physical function trajectories remained stable over time, as previously presented in the background Section [6]. Data from the CHECK cohort, that included people with early HKOA in 9-year follow-up with yearly assessments, concluded that two thirds of patients have an episode of pain or physical function deterioration, but overall, these outcomes maintained stable over time. Additionally, the authors found no association between the number of deterioration episodes and total joint replacement [27].

The Osteoarthritis Initiative cohort studies found similar stable course of HRQoL in people with knee OA, using the KOOS quality of life subscale [8, 28]. One of these studies included more than 3,000 people with mild knee OA (mild structural disease—Kellgren and Lawrence 2) that were followed yearly during 8-years. The authors additionally found a rapidly worsening HRQoL trajectory, that made up 9.5% of the participants and that these were at higher risk of total joint replacement [8]. Since we used 4 follow-ups in a 10-year period our data are not that sensible to detect patterns of change of rapidly worsening/improving trajectories. To our knowledge our study is the first that analyzed the trajectories of HRQoL of people with HKOA and including participants within the whole spectrum of the disease severity [6, 27].

Baseline differences in physical function and HRQoL, and corresponding subsequent trajectories assignment can be explained by the so-called “horse-racing effect”. This concept describes that the participants who have already started progressing in these outcomes, are likely to be “out in the front” (have worse physical function or HRQoL) at baseline, because they were already in a lower level of physical function or HRQoL before the start of the study, and will keep relative lower/higher levels of physical function and HRQoL through time [29]. This highlights the chronic nature of OA and slow decline of physical function and HRQoL in most people with HKOA. The horseracing effect has been previously described for other chronic health diseases in older adults [30].

In this study, modifiable and non-modifiable risk factors were consistently associated with moderate and low physical function and HRQoL trajectories. People with older age, female sex, presence of multimorbidity, high baseline clinical severity, and unmanageable pain levels were associated with low HRQoL and high disability trajectories, similarly to other longitudinal studies that analyzed the course of physical function [6, 31–34] and HRQoL [8, 28]. Other clinical important variables like unmanageable pain levels and BMI, despite their univariate association with the outcomes of this study, were excluded from the final multivariable model. Unmanageable pain levels showed a significant association with the “consistently moderate” HRQoL trajectory but not with any physical function trajectories. This conflicts with the literature, which shows that high pain intensity is an important predictor of HRQoL decline [8] and that pain explains most of the variability in disability and HRQoL [35]. On the other hand, higher clinical severity at baseline was associated with poorer physical function and HRQoL trajectories, as previously found in the literature [31]. Clinical severity, evaluated with HOOS/KOOS, encompasses several dimensions of OA consequences that fluctuate over time and are closely related to each other, such as pain, stiffness, and activity performance [36]. Therefore, symptoms conjunction and its consequences of activities performance may influence HKOA trajectories, being unmanageable pain levels (that differentiate people with more or less than 5 in NPRS) a less important factor when these variables are considered. Despite the importance of BMI, as a health-related behaviour variable, it was also excluded from the final model. Previous studies have showed that BMI is an important risk factor for OA onset [37] and severity [38]. However, the inclusion of other variables related with BMI like regular physical activity, multimorbidity and age may dilute the importance of BMI, as a single factor, on HRQoL and physical function trajectories.

We found that participants who reported regular physical activity at baseline had a lower risk of poor HRQoL and physical function trajectories, suggesting a protective effect on the symptoms and structural progression of OA [39]. In fact, maintaining regular physical activity is one of the core recommended interventions for HKOA [40] and most people follow improvement trajectories in physical function after physical activity programs [33, 34]. Therefore, we hypothesize that specific, personalized, and supportive interventions that consider modifiable risks factors for poor trajectories, such as physical activity, may improve HRQoL and physical function in the long term as well as the pattern of progression of these outcomes.

Multimorbidity was also included in the final model, showing significative associations with poorer physical function and HRQoL trajectories. Evidence shows that people with HKOA and multimorbidity, specifically cardiac diseases, hypertension, or back pain, are more likely to have worse physical function [41], mobility and mental health problems [42].

However, there is conflicting evidence for the association of older age with low HRQoL trajectories. Data from the Osteoarthritis Initiative showed that being younger was associated with lower quality of life (measured with KOOS quality of life subscale) in a population between 45 and 79 years old [28]. HRQoL loss in younger people may be explained by the impact of OA on work [43], whereas exposure to risk factors, structural changes and multimorbidity in older adults are also associated with functional and HRQoL decline [21, 44]. Because we used a generic measurement tool (EQ-5D-3L), our results may capture a broader image of HRQoL.

Previous literature corroborates our findings, suggest that female sex is associated with poor physical function [45] and HRQoL [8] trajectories, likely due to the gender gap in overall socioeconomic disadvantages of women, when compared to men. Additionally, women often report more activity limitations, multimorbidity, pain, depression, and self-reported health status when compared to men [22]. Therefore, the impact of OA in the long run may be different between the male and female sex [41, 42].

Since we found similar factors that predict the assignment of participants in physical function and HRQoL trajectories, we hypothesize that most patients that follow a progression of physical function decline may follow a similar HRQoL loss trajectoy. HRQoL is a more complex construct that goes beyond physical function, including also emotional and social well-being, corroborating the multidimensionality of HKOA impact [46]. Although our study did not show any association between anxiety or depression with HRQoL and physical function trajectories, previous literature shows that HRQoL in people with OA is influenced by gender, body weight, physical activity, mental health and education [46].

We examined the dimensions of physical function (HAQ) and HRQoL (EQ-5D-3L) in each follow-up evaluation and found poorer levels in the HAQ and in EQ-5D-3L dimensions in the second wave of the study (2013–2015) than in the other three waves. This was also reflected in the “consistently moderate” and “consistently low” HRQoL trajectories. The decrease of EQ-5D-3L levels in the second wave of this study may not be attributable only to the progression of HKOA. These results may be explained by the major economic crisis that occurred in Portugal at the time and led to high unemployment rates, lower monthly incomes, and consequent inequalities in access to healthcare [47]. These are factors known to be closely associated with health outcomes [48]. Regarding EQ-5D-3L, pain/discomfort, mobility, and usual activities were the most affected dimensions, highlighted previously as the dimensions with the most pronounced differences between people with OA and the general population [49]. Aligned with our results, a national study in Austria showed that the most impaired daily life activities for people with HKOA were heavy housework, bending or kneeling down, climbing stairs, and walking 500 m [50]. “Reach” was the activity in which a higher proportion of participants reported having difficulties, among HAQ domains. Palazzo et al. (2014) concluded that OA was the RMD with the major contribution to activity limitations, including lifting and carrying objects [51]. We did not control for other joint diseases, namely upper limb osteoarthritis or polyosteoarthritis, that may also decrease the capability to perform “reach” activities due to limitations on the upper limbs. However, in people with HKOA this may also reflect the decreased balance and lower limb strength [52, 53] that is needed to reach and lift objects from the floor or to reach objects above head.

### Limitations and strengths

This study is not free from limitations. First, only four waves were conducted for this cohort, and the last two were separated by a period of 5 years, which restricts the number of observations in the longitudinal analysis and may compromise the sensitivity of the trajectories. Studies with shorter time between measurements may capture a higher variability in the trajectories found, due to flares of pain and physical function loss, even if the trajectories are stable overall, in the long term. Baseline data collection occurred between 2011 and 2013, which placed individuals at different starting points and added variability that wasn’t accounted for. Second, there is a high proportion of participants included in the first phase of EpiDoC 1 that did not show up to clinical appointments and a high drop-out rate between participants with HKOA included in EpiDoC 1. We acknowledge the possibility of attrition bias since the individuals who were willing to participate in clinical appointments and the ones who continue to respond to follow-ups might be participants with higher HKOA clinical severity.

Our results should be interpreted taking into account the possibility of attrition bias between waves, since participants who dropped-out were older, had lower education and worse health outcomes overall. Despite the statistically significance differences between the included and drop-out participants, we did not find these differences as clinically relevant. Moreover, these variables have been previously associated with a higher risk of drop-out in longitudinal studies, however it is recognized that despite differences in some variables, the estimates of associations between variables can remain robust [54]. Therefore, we cautiously hypothesize that this may have influenced our results to a lower extent.

Moreover, we did not investigate differences between people with hip versus knee OA. Although OA of the hip and OA of the knee may impose similar burdens [2, 55], people with hip OA may have greater disease severity and an earlier requirement for joint replacement [56]. Lastly, we did not control other potential factors that may influence the classification into different HRQoL or physical function trajectory groups, namely psychosocial factors (e.g., coping strategies or self-efficacy) and interventions used. Furthermore, physical activity was self-reported, not taking into account the amount of time spent per week or the intensity; thus, our results may have overestimated the recommendations for physical activity.

However, this study used data from a large nationwide prospective cohort of adults from the community, and is not confined to people who seek healthcare. To the best of our knowledge, this is the first study to characterize trajectories of physical function and HRQoL among community adults with clinically validated HKOA using group-based trajectory modeling. This approach has been gaining momentum in the longitudinal analysis of clinical patient-reported outcomes, since it allows for the identification of unique subgroups of the population that follow distinct trajectories [57]. OA is a progressive chronic disease, a long follow-up period is needed to capture changes in health-outcomes. Few studies have follow-ups longer than 8 years, in opposite to our study [6].

This study shows, at a population-level and including a full spectrum of adults with HKOA from the community in terms of severity, with no age limits, that approximately 70% of people with HKOA follow stables trajectories of moderate/low physical function and HRQoL over 10 years, and that the trajectories of these two outcomes seem similar. Physical function and HRQoL trajectories are influenced by previously known factors from the literature, namely age, sex, physical activity, clinical severity, education and multimorbidity [6, 8].

Future research should validate HKOA outcome trajectories in a population-level in other contexts, analyze the interventions that may change trajectories (e.g., exercise, total joint replacement) and consider also the modifiable predictors of poor trajectories in the design of stratified interventions for people with HKOA.

## Conclusion

This study found three trajectories of physical function and HRQoL in a 10-year follow-up period. These trajectories were similar between the two outcomes and remained stable over time, with 70% of participants with HKOA maintaining moderate/low physical function and HRQoL levels. Female participants, multimorbidity, baseline high clinical severity, and unmanageable pain levels were positively associated with moderate/low HRQoL and physical function trajectories, whereas, high education level and baseline regular physical activity were protective. These risk factors may be considered in tailored OA multidisciplinary management programs that may target individuals’ modifiable risk factors of poor physical function and HRQoL trajectories, such as physical activity, clinical severity, pain levels and multimorbidity.

## Supplementary Information

**Additional file 1:** **Figure S1.** Percentagesof people with HKOA with at least somedifficulty (%) by HAQ domains at baseline (EpiDoC 1) and in each follow-up(EpiDoC 2, 3, and 4). **Figure S2.** Percentages of people with HKOA with at least one problem (%) by EQ-5D-3L dimensions at baseline (EpiDoC1) and in each follow-up (EpiDoC 2, 3, and 4). **Table S1.** Sociodemographic, lifestyles clinicalcharacteristics of HKOA participants in each EpiDoC wave. **Table S2.** Sociodemographic, lifestyles clinicalcharacteristics of HKOA participants by attrition status. **Table S3.** Average years to baseline in eachfollow-up wave of total sample and by trajectory group. **Table S4.** Frequencies of participants reportingat least one problem/difficulty divided by the total number of respondents, forEQ5D dimensions and HAQ Domains. **Table S5.**Bayesian Information Criterion (BIC) values and estimated group sizes (%). **Figure S3.** Trajectories forphysical function and HRQoL considering4 [a) and c)] and 5 [b) and d)] trajectory groups.**Table S6.** Trajectory model diagnostic criteria. **Table S7.** Univariate Multinomial Logistic Regression models for theassociation of baseline characteristics and physical function (HAQ)trajectories. **Table S8.**Univariate Multinomial Logistic Regression models for the association of HKOApatients baseline characteristics and HRQoL (EQ-5D) trajectories.

## Data Availability

The data underlying this article were provided by the EpiDoc Unit—CEDOC by permission. Data will be shared upon request to the corresponding author with the permission of EpiDoc Unit group leaders.

## Abbreviations

BIC: Bayesian Information Criteria
BMI: Body mass index
EQ-5D-3L: EuroQoL, 5-dimension and 3-level
HAQ: Health Assessment Questionnaire
HKOA: Hip and/or Knee Osteoarthritis
HRQoL: Health-Related Quality of Life
KOOS: Knee Injury and Osteoarthritis Outcome Score
NPRS: Numeric Pain Rating Scale
NUTS II: Nomenclature of Territorial Units for Statistical purposes, *Nomenclatura das Unidades Territoriais para Fins Estatísticos*
OA: Osteoarthritis
RRR: Relative Risk Ratio

## References

[1] Global, regional, and national incidence, prevalence, and years lived with disability for 310 diseases and injuries, 1990–2015: a systematic analysis for the Global Burden of Disease Study 2015. Lancet. 2016;388(10053):1545–602. Available from: https://www.ncbi.nlm.nih.gov/pubmed/27733282 10.1016/S0140-6736(16)31678-6 PMC5055577 27733282

[2] Safiri S, Kolahi AA, Smith E, Hill C, Bettampadi D, Mansournia MA (2020). Global, regional and national burden of osteoarthritis 1990–2017: a systematic analysis of the Global Burden of Disease Study 2017. Ann Rheum Dis.

[3] Salmon JH, Rat AC, Sellam J, Michel M, Eschard JP, Guillemin F (2016). Economic impact of lower-limb osteoarthritis worldwide: a systematic review of cost-of-illness studies. Osteoarthr Carti.

[4] Hawker GA (2019). Osteoarthritis is a serious disease. Clin Exp Rheumatol.

[5] Dell’Isola A, Allan R, Smith SL, Marreiros SS, Steultjens M (2016). Identification of clinical phenotypes in knee osteoarthritis: a systematic review of the literature. BMC Musculoskelet Disord.

[6] Wieczorek M, Rotonda C, Guillemin F, Rat A (2020). What Have We Learned From Trajectory Analysis of Clinical Outcomes in Knee and Hip Osteoarthritis Before Surgery?. Arthritis Care Res (Hoboken).

[7] Hunter DJ, Nicolson PJA, Little CB, Robbins SR, Wang X, Bennell KL (2019). Developing strategic priorities in osteoarthritis research: Proceedings and recommendations arising from the 2017 Australian Osteoarthritis Summit. BMC Musculoskelet Disord.

[8] Törmälehto S, Aarnio E, Mononen ME, Arokoski JPA, Korhonen RK, Martikainen JA (2019). Eight-year trajectories of changes in health-related quality of life in knee osteoarthritis: Data from the Osteoarthritis Initiative (OAI). PLoS One..

[9] Rodrigues AM, Gouveia N, da Costa LP, Eusébio M, Ramiro S, Machado P (2015). EpiReumaPt- the study of rheumatic and musculoskeletal diseases in Portugal: a detailed view of the methodology. Acta Reumatol Port.

[10] Laires PA, Canhao H, Araujo D, Fonseca JE, Machado P, Mourao AF, Ramiro S, et al. CoReumaPt protocol: the Portuguese cohort of rheumatic diseases. Acta Reumatol Port. 2012;37(1):18–24. 22781511

[11] Brown C, Cooke TD, Daniel W, Greenwald RGR, Hochberg M, Howell D (1990). Arthritis & Rheumatism The American College of Rheumatology reporting of osteoarthritis of the hand. Arthritis Rheum..

[12] Altman R, Asch E, Bloch D, Bole G, Borenstein D, Brandt K (1986). Development of criteria for the classification and reporting of osteoarthritis: Classification of osteoarthritis of the knee. Arthritis Rheum..

[13] Fries JF, Spitz P, Kraines RG, Holman HR (1980). Measurement of patient outcome in arthritis. Arthritis Rheum.

[14] Ferreira LN, Ferreira PL, Pereira LN, Oppe M (2014). The valuation of the EQ-5D in Portugal. Qual Life Res.

[15] Diederichs C, Berger K, Bartels DB (2011). The Measurement of Multiple Chronic Diseases–A Systematic Review on Existing Multimorbidity Indices. J Gerontol A Biol Sci Med Sci..

[16] Goncalves RS, Cabri J, Pinheiro JP, Ferreira PL, Gil J. Reliability, validity and responsiveness of the Portuguese version of the Knee injury and Osteoarthritis Outcome Score--Physical Function Short-form (KOOS-PS). Osteoarthr Cartil. 2010;18(3):372–6. 10.1016/j.joca.2009.10.012 19912982

[17] Cavalheiro L, Gil J, Nunes S, Ferreira P, Gonçalves R. Measuring Health-Related Quality of Life in Patients With Hip Osteoarthritis and Total Hip Replacement: Adaption and Validation of the Hip Disability and Osteoarthritis Outcome Source LK 2.0 (HOOS 2.0) to the Portuguese Culture. In: 18th Annual Conference of the International Society of Quality of Life (ISOQOL 2011). 2011; 20:79–79.

[18] Roos EM, Engelhart L, Ranstam J, Anderson AF, Irrgang JJ, Marx RG (2011). ICRS recommendation document: Patient-reported outcome instruments for use in patients with articular cartilage defects. Cartilage.

[19] Brisson NM, Gatti AA, Maly MR (2020). Association of Pain and Steps Per Day in Persons With Mild-to-Moderate, Symptomatic Knee Osteoarthritis: A Mixed-Effects Models Analysis of Multiple Measurements Over Three Years. Arthritis Care Res (Hoboken).

[20] Zelman DC, Hoffman DL, Seifeldin R, Dukes EM (2003). Development of a metric for a day of manageable pain control: Derivation of pain severity cut-points for low back pain and osteoarthritis. Pain.

[21] Hunter DJ, Bierma-Zeinstra S (2019). Osteoarthritis. Lancet.

[22] Schmitz A, Lazarevič P (2020). The gender health gap in Europe’s ageing societies: universal findings across countries and age groups?. Eur J Ageing.

[23] Nagin DS, Odgers CL (2010). Group-based trajectory modeling in clinical research. Annu Rev Clin Psychol.

[24] Craig MA, Zou LX, Bai H, Lee MM. Stereotypes About Political Attitudes and Coalitions Among U.S. Racial Groups: Implications for Strategic Political Decision-Making. Pers Soc Psychol Bull. 2022; 48(9):1349–66. 10.1177/01461672211037134 34384287

[25] James G, Witten D, Hastie T, Tibshirani R. An Introduction to Statistical Learning: With Applications in R. 7th printed edtion. New York: Springer; 2017.

[26] Fagerland MW, Hosmer DW (2012). A Generalized Hosmer-Lemeshow Goodness-of-Fit Test for Multinomial Logistic Regression Models. Stata J.

[27] Gademan MGJ, Putter H, Van Den Hout WB, Kloppenburg M, Hofstede SN, Cannegieter SC (2018). The course of pain and function in osteoarthritis and timing of arthroplasty: the CHECK cohort. Acta Orthop.

[28] Han A, Gellhorn AC (2018). Trajectories of Quality of Life and Associated Risk Factors in Patients With Knee Osteoarthritis. Am J Phys Med Rehabil.

[29] Peto R (1981). The horse-racing effect. Lancet.

[30] Cesari M, Canevelli M (2014). Horse-racing effect and clinical trials in older persons. Front Aging Neurosci.

[31] Bastick AN, Runhaar J, Belo JN, Bierma-Zeinstra SMA. Prognostic factors for progression of clinical osteoarthritis of the knee: a systematic review of observational studies. Arthritis Res Ther. 2015;17(1). Available from: http://arthritis-research.com/content/17/1/152 10.1186/s13075-015-0670-x PMC4483213 26050740

[32] White DK, Neogi T, Nguyen USDT, Niu J, Zhang Y (2016). Trajectories of functional decline in knee osteoarthritis: the Osteoarthritis Initiative. Rheumatology.

[33] Berg B, Roos EM, Kise NJ, Engebretsen L, Holm I, Risberg MA (2021). On a Trajectory for Success—9 in Every 10 People With a Degenerative Meniscus Tear Have Improved Knee Function Within 2 Years After Treatment: A Secondary Exploratory Analysis of a Randomized Controlled Trial. J Orthop Sports Phys Ther.

[34] Lee AC, Harvey WF, Han X, Price LL, Driban JB, Bannuru RR (2018). Pain and functional trajectories in symptomatic knee osteoarthritis over up to 12 weeks of exercise exposure. Osteoarthr Cartil.

[35] Montero A, Mulero JF, Tornero C, Guitart J, Serrano M (2016). Pain, disability and health-related quality of life in osteoarthritis—joint matters: an observational, multi-specialty trans-national follow-up study. Clin Rheumatol.

[36] O’Neill TW, McCabe PS, McBeth J (2018). Update on the epidemiology, risk factors and disease outcomes of osteoarthritis. Best Pract Res Clin Rheumatol.

[37] Silverwood V, Blagojevic-Bucknall M, Jinks C, Jordan JL, Protheroe J, Jordan KP (2015). Current evidence on risk factors for knee osteoarthritis in older adults: a systematic review and meta-analysis. Osteoarthr Cartil..

[38] Deveza LA, Melo L, Yamato TP, Mills K, Ravi V, Hunter DJ (2017). Knee osteoarthritis phenotypes and their relevance for outcomes: a systematic review. Osteoarthr Cartil.

[39] Raud B, Gay C, Guiguet-Auclair C, Bonnin A, Gerbaud L, Pereira B (2020). Level of obesity is directly associated with the clinical and functional consequences of knee osteoarthritis. Sci Rep.

[40] Bannuru RR, Osani MC, Vaysbrot EE, Arden NK, Bennell K, Bierma-Zeinstra SMA, et al. OARSI guidelines for the non-surgical management of knee, hip, and polyarticular osteoarthritis. Osteoarthr Cartil. 2019;27(11):1578–89. 10.1016/j.joca.2019.06.011 31278997

[41] Calders P, Van Ginckel A (2018). Presence of comorbidities and prognosis of clinical symptoms in knee and/or hip osteoarthritis: a systematic review and meta-analysis. Semin Arthritis Rheum.

[42] Muckelt PE, Roos E, Stokes M, McDonough S, Grønne D, Ewings S (2020). Comorbidities and their link with individual health status: a cross-sectional analysis of 23,892 people with knee and hip osteoarthritis from primary care. J Comorb.

[43] Ackerman IN, Bucknill A, Page RS, Broughton NS, Roberts C, Cavka B (2015). The substantial personal burden experienced by younger people with hip or knee osteoarthritis. Osteoarthr Cartil.

[44] Ofori-Asenso R, Chin KL, Curtis AJ, Zomer E, Zoungas S, Liew D (2019). Recent patterns of multimorbidity among older adults in high-income countries. Popul Health Manag.

[45] Wieczorek M, Rotonda C, Coste J, Pouchot J, Saraux A, Guillemin F (2020). Trajectory analysis combining pain and physical function in individuals with knee and hip osteoarthritis: results from the French KHOALA cohort. Rheumatology.

[46] Verges J, Vitaloni M, Bibas M, Sciortino R, Quintero M, Monfort J (2019). Global oa management begins with quality of life assessment in knee oa patients: a systematic review. Osteoarthr Cartil.

[47] Macassa G, Rodrigues C, Barros H, Marttila A (2021). Experiences of involuntary job loss and health during the economic crisis in Portugal. Porto Biomed J.

[48] Simou E, Koutsogeorgou E (2014). Effects of the economic crisis on health and healthcare in Greece in the literature from 2009 to 2013: a systematic review. Health Policy (New York).

[49] Martín-Fernández J, García-Maroto R, Bilbao A, García-Pérez L, Gutiérrez-Teira B, Molina-Siguero A (2020). Impact of lower limb osteoarthritis on healthrelated quality of life: A cross-sectional study to estimate the expressed loss of utility in the Spanish population. PLoS One.

[50] Stamm TA, Pieber K, Crevenna R, Dorner TE. Impairment in the activities of daily living in older adults with and without osteoporosis, osteoarthritis and chronic back pain: A secondary analysis of population-based health survey data. BMC Musculoskelet Disord. 2016;17(1). 10.1186/s12891-016-0994-y PMC4810518 27020532

[51] Palazzo C, Ravaud JF, Papelard A, Ravaud P, Poiraudeau S (2014). The Burden of Musculoskeletal Conditions. PLoS One.

[52] Wang DXM, Yao J, Zirek Y, Reijnierse EM, Maier AB (2020). Muscle mass, strength, and physical performance predicting activities of daily living: a meta-analysis. J Cachexia Sarcopenia Muscle.

[53] Hislop A, Collins NJ, Tucker K, Semciw AI (2022). Hip strength, quadriceps strength and dynamic balance are lower in people with unilateral knee osteoarthritis compared to their non-affected limb and asymptomatic controls. Braz J Phys Ther.

[54] Gustavson K, von Soest T, Karevold E, Røysamb E (2012). Attrition and generalizability in longitudinal studies: findings from a 15-year population-based study and a Monte Carlo simulation study. BMC Public Health.

[55] Postler A, Ramos AL, Goronzy J, Gunther KP, Lange T, Schmitt J (2018). Prevalence and treatment of hip and knee osteoarthritis in people aged 60 years or older in Germany: an analysis based on health insurance claims data. Clin Interv Aging..

[56] Dabare C, Le Marshall K, Leung A, Page CJ, Choong PF, Lim KK (2017). Differences in presentation, progression and rates of arthroplasty between hip and knee osteoarthritis: Observations from an osteoarthritis cohort study-a clear role for conservative management. Int J Rheum Dis.

[57] Nagin DS (2014). Group-Based Trajectory Modeling: An Overview. Ann Nutr Metab.

