# Supplementary material for: Trajectories of physical function and quality of life in people with osteoarthritis: results from a 10-year population-based cohort
Source: BMC Public Health. 2023 Jul 21;23:1407. doi: 10.1186/s12889-023-16167-9 (PMC10362599; doi:10.1186/s12889-023-16167-9)
Supplement: Supplementary file 1 — Additional file 1: Figure S1. Percentagesof people with HKOA with at least somedifficulty (%) by HAQ domains at baseline (EpiDoC 1) and in each follow-up(EpiDoC 2, 3, and 4). Figure S2. Percentages of people with HKOA with at least one problem (%) by EQ-5D-3L dimensions at baseline (EpiDoC1) and in each follow-up (EpiDoC 2, 3, and 4). Table S1. Sociodemographic, lifestyles clinicalcharacteristics of HKOA participants in each EpiDoC wave. Table S2. Sociodemographic, lifestyles clinicalcharacteristics of HKOA participants by attrition status. Table S3. Average years to baseline in eachfollow-up wave of total sample and by trajectory group. Table S4. Frequencies of participants reportingat least one problem/difficulty divided by the total number of respondents, forEQ5D dimensions and HAQ Domains. Table S5.Bayesian Information Criterion (BIC) values and estimated group sizes (%). Figure S3. Trajectories forphysical function and HRQoL considering4 [a) and c)] and 5 [b) and d)] trajectory groups.Table S6. Trajectory model diagnostic criteria. Table S7. Univariate Multinomial Logistic Regression models for theassociation of baseline characteristics and physical function (HAQ)trajectories. Table S8.Univariate Multinomial Logistic Regression models for the association of HKOApatients baseline characteristics and HRQoL (EQ-5D) trajectories. [file 12889_2023_16167_MOESM1_ESM.docx]

**Additional File 1**

**Figure S1**

**Figure S1**. Percentages of people with HKOA with at least some difficulty (%) by HAQ domains at baseline (EpiDoC 1) and in each follow-up (EpiDoC 2, 3, and 4).

**Figure S2**

**Figure S2**. Percentages of people with HKOA with at least one problem (%) by EQ-5D-3L dimensions at baseline (EpiDoC 1) and in each follow-up (EpiDoC 2, 3, and 4).

**Table S1**. Sociodemographic, lifestyles clinical characteristics of HKOA participants in each EpiDoC wave

|  | EpiDoC 1  (2011-2013)  n=983 | EpiDoC 2  (2013-2015)  n=969 | EpiDoC 3  (2015-2016)  n=749 | EpiDoC 4  (2021)  n=453 |
| --- | --- | --- | --- | --- |
| Sociodemographic |  |  |  |  |
| Sex, n(%) |  |  |  |  |
| Female | 701 (71.3%) | 692 (71.4%) | 538 (71.8%) | 332 (73.3%) |
| Age (years old) |  |  |  |  |
| Mean (SD) | 65.2 (11.2) | 66.3 (11.2) | 67.3 (11.5) | 71.6 (11.0) |
| <55 years old, n (%) | 170 (17.3%) | 155 (16.0%) | 102 (13.6%) | 26 (5.7%) |
| 55-64 years old, n (%) | 265 (27.0%) | 245 (25.3%) | 177 (23.6%) | 89 (19.6%) |
| 65-74 years old, n (%) | 340 (34.6%) | 328 (33.8%) | 245 (32.7%) | 144 (31.8%) |
| ³75 years old, n (%) | 208 (21.2%) | 241 (24.9%) | 225 (30.0%) | 194 (42.8%) |
| Region (NUTSII), n (%) |  |  |  |  |
| North | 267 (27.2%) | 264 (27.2%) | 195 (26.0%) | 125 (27.6%) |
| Centre | 238 (24.2%) | 233 (24.0%) | 175 (23.4%) | 107 (23.6%) |
| Lisbon | 165 (26.8%) | 163 (16.8%) | 122 (16.3%) | 91 (20.1%) |
| Alentejo | 58 (5.9%) | 57 (5.9%) | 48 (6.4%) | 22 (4.9%) |
| Algarve | 20 (2.0%) | 20 (2.1%) | 15 (2.0%) | 11 (2.4%) |
| Islands | 235 (23.9%) | 232 (23.9%) | 194 (25.9%) | 97 (21.4%) |
| Marital status, n (%) |  |  |  |  |
| With partner | 641 (65.2%) | 631 (65.1%) | 492 (65.7%) | 313 (69.1%) |
| Educational Level, n (%) |  |  |  |  |
| <4 years | 230 (23.4%) | 225 (23.2%) | 167 (22.3%) | 87 (19.2%) |
| 4-9 years | 630 (64.1%) | 621 (64.1%) | 488 (65.1%) | 293 (64.7%) |
| ³10 years | 123 (12.5%) | 123 (12.7%) | 94 (12.6%) | 73 (16.1%) |
| Anthropometric |  |  |  |  |
| BMI (kg/m^2^), n (%) | n=919 | n=891 | n=664 | n=399 |
| Underweight/Normal weight | 168 (18.3%) | 177 (19.9%) | 137 (20.6%) | 82 (20.6%) |
| Overweight | 376 (40.9%) | 377 (42.3%) | 311 (46.8%) | 168 (42.1%) |
| Obese | 375 (40.8%) | 337 (37.8%) | 216 (32.5%) | 149 (37.3%) |
| Lifestyle |  |  |  |  |
| Smoking habits n (%) |  |  |  |  |
| Never | 732 (74.5%) | 700 (73.9%) | 551 (75.3%) | 331 (77.0%) |
| In the past | 180 (18.3%) | 187 (19.8%) | 133 (18.2%) | 76 (17.7%) |
| Daily/Occasionally | 71 (7.2%) | 60 (6.3%) | 48 (6.6%) | 23 (5.3%) |
| Alcohol consumption n (%) |  |  |  |  |
| Never | 497 (50.6%) | 546 (57.7%) | 324 (44.2%) | 224 (52.1%) |
| Occasionally/Daily | 485 (49.4%) | 401 (42.3%) | 409 (55.8%) | 206 (47.9%) |
| Regular physical activity  n (%) |  |  |  |  |
| Yes | 212 (21.6%) | 374 (39.1%) | 270 (36.8%) | 159 (37.0%) |
| Clinical |  |  |  |  |
| Multimorbidity  n (%) |  |  |  |  |
| Yes | 634 (70.8%) | 627 (72.9%) | 501 (78.5%) | 297 (74.6%) |
| Clinical severity  (inverted HOOS/KOOS score) |  |  |  |  |
| Mean (SD) | 46.1 (18.8) | - | - | - |
| Unmanageable pain levels  (≥5 NPRS), n (%) |  |  |  |  |
| Yes | 691 (73.8%) | - | - | - |
| Physical function  (HAQ score) – Mean (SD) | 0.8 (0.7) | 1.0 (0.7) | 0.9 (0.7) | 0.9 (0.7) |
| HRQoL  (EQ5D score) – Mean (SD) | 0.6 (0.3) | 0.5 (0.3) | 0.6 (0.3) | 0.6 (0.3) |

Sample size is not constant due to missing values in some variables: BMI – EpiDoC1 (n=919), EpiDoC2 (n=891), EpiDoC3 (n=664), EpiDoC4 (n=399); Smoking habits – EpiDoC2 (n=947), EpiDoC3 (n=732), EpiDoC4 (n=430); Alcohol consumption – EpiDoC1 (n=982), EpiDoC2 (n=947), EpiDoC3 (n=733), EpiDoC4 (n=430); Regular physical activity – EpiDoC1 (n=982), EpiDoC2 (n=957), EpiDoC3 (n=734), EpiDoC4 (n=430); Multimorbidity – EpiDoC1 (n=895), EpiDoC2 (n=860), EpiDoC3 (n=638), EpiDoC4 (n=398); Clinical severity – EpiDoC1 (n=903); Unmanageable pain levels – EpiDoC1 (n=936); Physical function (HAQ) – EpiDoC1 (n=983), EpiDoC2 (n=954), EpiDoC3 (n=729), EpiDoC4 (n=408); HRQoL (EQ-5D-3L) – EpiDoC1 (n=971), EpiDoC2 (n=951), EpiDoC3 (n=720), EpiDoC4 (n=405).

**Table S2**. Sociodemographic, lifestyles clinical characteristics of HKOA participants by attrition status.

|  | EpiDoC 2  out  n=14 | EpiDoC 2  in  n=969 | *p* | EpiDoC 3  out  n=234 | EpiDoC 3  in  n=749 | *p* | EpiDoC 4  out  n=530 | EpiDoC 4  in  n=453 | *p* |
| --- | --- | --- | --- | --- | --- | --- | --- | --- | --- |
| Sociodemographic |  |  |  |  |  |  |  |  |  |
| Sex, n(%) |  |  | 0.558 |  |  | 0.522 |  |  | 0.205 |
| Female | 9 (64.3%) | 692 (71.4%) |  | 163 (69.7%) | 538 (71.8%) |  | 369 (69.6%) | 332 (73.3%) |  |
| Age (years old) |  |  |  |  |  |  |  |  |  |
| Mean (SD) | 72.8 (9.5) | 65.1 (11.2) | 0.006 | 68.3 (10.2) | 65.7 (11.5) | 0.007 | 69.4 (11.9) | 65.6 (10.8) | <0.001 |
| Region (NUTSII), n (%) |  |  | - |  |  | 0.091 |  |  | 0.075 |
| North | 3 (21.4%) | 264 (27.2%) |  | 72 (30.8%) | 195 (26.0%) |  | 142 (26.8%) | 125 (27.6%) |  |
| Centre | 5 (35.7%) | 233 (24.1%) |  | 63 (26.9%) | 175 (23.4%) |  | 131 (24.7%) | 107 (23.6%) |  |
| Lisbon | 2 (14.3%) | 163 (16.8%) |  | 43 (18.4%) | 122 (16.3%) |  | 74 (14.0%) | 91 (20.1%) |  |
| Alentejo | 1 (7.1%) | 57 (5.9%) |  | 10 (4.3%) | 48 (6.4%) |  | 36 (6.8%) | 22 (4.9%) |  |
| Algarve | - | 20 (2.1%) |  | 5 (2.1%) | 15 (2.0%) |  | 9 (1.7%) | 11 (2.4%) |  |
| Islands | 3 (21.4%) | 232 (23.9%) |  | 41 (17.5%) | 194 (25.9%) |  | 138 (26.0%) | 97 (21.4%) |  |
| Marital status, n (%) |  |  | 0.623 |  |  | 0.573 |  |  | 0.018 |
| With partner | 10 (71.4%) | 631 (65.1%) |  | 149 (63.7%) | 492 (65.7%) |  | 328 (61.9%) | 313 (69.1%) |  |
| Educational Level, n (%) |  |  | 0.260 |  |  | 0.334 |  |  | 0.001 |
| <4 years | 5 (35.7%) | 225 (23.2%) |  | 63 (26.9%) | 167 (22.3%) |  | 143 (27.0%) | 87 (19.2%) |  |
| 4-9 years | 9 (64.3%) | 621 (64.1%) |  | 142 (60.7%) | 488 (65.1%) |  | 337 (63.6%) | 293 (64.7%) |  |
| ³10 years | - | 123 (12.7%) |  | 29 (12.4%) | 94 (12.6%) |  | 50 (9.4%) | 73 (16.1%) |  |
| Anthropometric |  |  |  |  |  |  |  |  |  |
| BMI (kg/m^2^), n (%) |  |  | 0.257 |  |  | 0.174 |  |  | 0.058 |
| Underweight/Normal weight | - | 168 (18.5%) |  | 49 (24.5%) | 128 (18.5%) |  | 69 (24.2%) | 68 (17.9%) |  |
| Overweight | 6 (50.0%) | 370 (40.8%) |  | 79 (39.5%) | 298 (43.1%) |  | 120 (42.1%) | 191 (50.4%) |  |
| Obese | 6 (50.0%) | 369 (40.7%) |  | 72 (36.0%) | 265 (38.4%) |  | 96 (33.7%) | 120 (31.7%) |  |
| Lifestyle |  |  |  |  |  |  |  |  |  |
| Smoking habits n (%) |  |  | - |  |  | 0.916 |  |  | 0.423 |
| Never | 12 (85.7%) | 720 (74.3%) |  | 162 (73.0%) | 538 (74.2%) |  | 239 (73.1%) | 312 (77.0%) |  |
| In the past | 2 (14.3%) | 178 (18.4%) |  | 46 (20.7%) | 141 (19.5%) |  | 66 (20.2%) | 67 (16.5%) |  |
| Daily/Occasionally | - | 71 (7.3%) |  | 14 (6.3%) | 46 (6.3%) |  | 22 (6.7%) | 26 (6.4%) |  |
| Alcohol consumption n (%) |  |  | 0.622 |  |  | 0.493 |  |  | 0.157 |
| Never | 8 (57.1%) | 489 (50.5%) |  | 133 (59.6%) | 413 (57.0%) |  | 154 (47.1%) | 170 (41.9%) |  |
| Occasionally/Daily | 6 (42.9%) | 479 (49.5%) |  | 90 (40.4%) | 311 (43.0%) |  | 173 (52.9%) | 236 (58.1%) |  |
| Regular physical activity  n (%) |  |  | 0.522 |  |  | 0.867 |  |  | 0.239 |
| Yes | 4 (28.6%) | 208 (21.5%) |  | 89 (39.6%) | 285 (38.9%) |  | 113 (34.5%) | 157 (38.7%) |  |
| Clinical |  |  |  |  |  |  |  |  |  |
| Multimorbidity  n (%) |  |  | 0.749 |  |  | 0.103 |  |  | 0.025 |
| Yes | 9 (75.0%) | 625 (70.8%) |  | 160 (77.3%) | 467 (71.5%) |  | 233 (82.6%) | 268 (75.3%) |  |
| Clinical severity  (inverted HOOS/KOOS score) |  |  | 0.328 |  |  | 0.001 |  |  | 0.005 |
| Mean (SD) | 50.9 (17.0) | 46.0 (18.8) |  | 49.8 (18.2) | 45.0 (18.8) |  | 47.7 (18.5) | 44.2 (19.0) |  |
| Unmanageable pain levels  (≥5 NPRS), n (%) |  |  | 0.439 |  |  | 0.811 |  |  | 0.847 |
| Yes | 7 (63.6%) | 684 (74.0%) |  | 166 (74.4%) | 525 (73.6%) |  | 373 (73.6%) | 318 (74.1%) |  |
| Physical function  (HAQ score) – Mean (SD) | 1.11 (0.86) | 0.74 (0.67) | 0.107 | 1.05 (0.72) | 0.96 (0.66) | 0.177 | 0.96 (0.75)be | 0.76 (0.65) | <0.001 |
| HRQoL  (EQ5D score) – Mean (SD) | 0.52 (0.32) | 0.62 (0.27) | 0.311 | 0.41 (0.30) | 0.48 (0.27) | 0.001 | 0.53 (0.32) | 0.59 (0.30) | 0.013 |

*p* – *p*-value from Chi-square tests for categorical variables or Wilcoxon rank-sum test for continuous variables. Chi-square testes were not performed for tables with >20% expected cell count less than 5. Tests assumed variable information from the previous common wave as available, e.g., age compared between EpiDoC 3 out and EpiDoC 3 in groups was the age at EpiDoC 2. Sex, region, marital status, education level, clinical severity and unmanageable pain levels were only collected and tested for baseline

**Table S3**. Average years to baseline in each follow-up wave of total sample and by trajectory group

|  |  | HRQoL (EQ-5D score) | | | | Physical Function (HAQ score) | | | |
| --- | --- | --- | --- | --- | --- | --- | --- | --- | --- |
|  | **Total Sample**  n=983 | **Consistently high HRQoL**  n=165 | **Consistently moderate HRQoL**  n=501 | **Consistently low HRQoL**  n=317 | **p^a^** | **Stable high disability**  n=204 | **Slightly worsening moderate disability**  n=472 | **Consistently low disability**  n=307 | **p^a^** |
| Years to baseline  Mean (SD) |  |  |  |  |  |  |  |  |  |
| EpiDoC 1 | **-** | **-** | **-** | **-** | **-** | **-** | **-** | **-** | **-** |
| EpiDoC 2 | 1.3 (0.6) | 1.4 (0.7) | 1.4 (0.6) | 1.3 (0.6) | 0.051 | 1.3 (0.6) | 1.3 (0.6) | 1.4 (0.6) | 0.004 |
| EpiDoC 3 | 2.8 (0.7) | 2.7 (0.7) | 2.8 (0.6) | 2.8 (0.7) | 0.164 | 2.8 (0.7) | 2.8 (0.6) | 2.9 (0.7) | 0.008 |
| EpiDoC 4 | 8.4 (0.6) | 8.4 (0.7) | 8.5 (0.6) | 8.3 (0.6) | 0.688 | 8.3 (0.6) | 8.3 (0.6) | 8.5 (0.6) | 0.203 |
| ^a^ *p*-values for Kruskal–Wallis non-parametric independency test | | | | | | | | | |

**Table S4**. Frequencies of participants reporting at least one problem/difficulty divided by the total number of respondents, for EQ5D dimensions and HAQ Domains.

| **EQ5D** | **EpiDoC 1** | **EpiDoC 2** | **EpiDoC 3** | **EpiDoC 4** |
| --- | --- | --- | --- | --- |
| Mobility | 528/983 (53.7%) | 676/955 (70.79%) | 387/731 (52.94%) | 195/409 (47.7%) |
| Selfcare | 194/983 (19.7%) | 356/957 (37.2%) | 260/731 (35.57%) | 125/409 (30.6%) |
| Activities | 373/981 (38.0%) | 535/957 (55.90%) | 317/731 (43.47%) | 163/408 (40.0%) |
| Pain | 602/983 (61.2%) | 715/955 (74.87%) | 442/731 (60.47%) | 260/410 (63.4%) |
| Anxiety/  Depression | 296/973 (30.4%) | 538/950 (57.68%) | 294/720 (40.83%) | 99/410 (24.2%) |
| **HAQ** | **EpiDoC 1** | **EpiDoC 2** | **EpiDoC 3** | **EpiDoC 4** |
| Dressing & Grooming | 415/983 (42.2%) | 567/953 (59.5%) | 395/728 (54.3%) | 199/408 (48.8%) |
| Arising | 532/983 (54.1%) | 655/953 (68.7%) | 431/728 (59.2%) | 228/407 (56.0%) |
| Eating | 334/983 (34.0%) | 387/951 (40.7%) | 246/728 (33.8%) | 156/406 (38.4%) |
| Walking | 505/983 (51.4%) | 672/952 (70.6%) | 460/728 (63.2%) | 262/407 (64.4%) |
| Hygiene | 387/983 (39.4%) | 445/952 (46.7%) | 312/727 (42.9%) | 182/402 (45.3%) |
| Reach | 677/983 (68.9%) | 739/946 (78.1%) | 533/728 (73.2%) | 277/407 (68.0%) |
| Grip | 295/983 (30.0%) | 325/949 (34.3%) | 228/728 (31.3%) | 124/404 (30.7%) |
| Activities | 373/981 (38.0%) | 535/957 (55.9%) | 317/731 (43.4%) | 163/408 (40.0%) |

**Table S5**. Bayesian Information Criterion (BIC) values and estimated group sizes (%).

| **Number of groups** | **Polynomial order^a^** | **BIC** | **2(ΔBIC)** | **Evidence against H0** | **Estimated group sizes** | | | | |
| --- | --- | --- | --- | --- | --- | --- | --- | --- | --- |
| **EQ5D** | | |  |  |  |  |  |  |  |
| 2 | 22 | -1458.18 |  |  | 66.3 | 33.7 |  |  |  |
| 3 | 222 | -1440.05 | 36.3 | Very strong | 33.5 | 48.3 | 18.2 |  |  |
| 4 | 2222 | -1367.79 | 144.5 | Very strong | 28.2 | 46.0 | 10.2 | 15.5 |  |
| 5 | 22222 | -1349.87 | 35.8 | Very strong | 27.1 | 40.9 | 9.9 | 7.2 | 15.0 |
| 3 (final) | 221 | -1436.64 |  |  | 33.4 | 48.4 | 18.3 |  |  |
| **HAQ** | | |  |  |  |  |  |  |  |
| 2 | 22 | -3211.63 |  |  | 60.7 | 39.3 |  |  |  |
| 3 | 222 | -3098.29 | 226.7 | Very strong | 30.2 | 47.5 | 22.3 |  |  |
| 4 | 2222 | -3077.43 | 41.7 | Very strong | 18.8 | 43.5 | 27.7 | 10.0 |  |
| 5 | 22222 | -3080.47 | -6.1 | Not worth mentioning | 12.4 | 32.5 | 29.7 | 18.9 | 6.4 |
| 3 (final) | 210 | -3099.59 |  |  | 32.0 | 47.0 | 21.0 |  |  |

**^a^** Order shape: 0–intercept, 1–linear, 2–quadratic, 3-cubic; 2(ΔBIC) – log of the Bayes factor approximation; By BIC criterion alone, the optimal number of groups for EQ5D was 5, and for HAQ was 4. However, due to sample size in each trajectory and to optimize clinical interpretation the decision to keep the models with three groups was made.

a)

a)b)

c) d)

**Figure S3.** Trajectories for physical function and HRQoL considering 4 [a) and c)] and 5 [b) and d)] trajectory groups.

**Table S6**. Trajectory model diagnostic criteria.

|  | **n** | **Estimated group probabilities** | **Proportion classified^a^** | **APP^b^** | **OCC^c^** |
| --- | --- | --- | --- | --- | --- |
|  |  | **3 groups** |  |  |  |
| **EQ5D Group** |  |  |  |  |  |
| Consistently low | 317 | 0.322 | 0.333 | 0.793 | 8.038 |
| Consistently moderate | 501 | 0.509 | 0.484 | 0.759 | 3.026 |
| Consistently high | 165 | 0.168 | 0.183 | 0.817 | 22.201 |
| **HAQ Group** |  |  |  |  |  |
| Consistently low disability | 307 | 0.312 | 0.320 | 0.876 | 15.500 |
| Slight worsening moderate disability | 472 | 0.480 | 0.470 | 0.848 | 6.056 |
| Consistently high disability | 204 | 0.208 | 0.210 | 0.883 | 28.935 |
|  |  | 4 groups |  |  |  |
| **EQ5D Group** |  |  |  |  |  |
| 1 | 266 | 0.271 | 0.282 | 0.786 | 9.915 |
| 2 | 475 | 0.483 | 0.460 | 0.762 | 3.431 |
| 3 | 89 | 0.091 | 0.102 | 0.764 | 32.459 |
| 4 | 153 | 0.156 | 0.155 | 0.759 | 17.045 |
| **HAQ Group** |  |  |  |  |  |
| 1 | 171 | 0.174 | 0.188 | 0.844 | 25.703 |
| 2 | 454 | 0.461 | 0.435 | 0.808 | 4.915 |
| 3 | 267 | 0.272 | 0.277 | 0.798 | 10.619 |
| 4 | 91 | 0.093 | 0.100 | 0.861 | 61.096 |
|  |  | 5 groups |  |  |  |
| **EQ5D Group** |  |  |  |  |  |
| 1 | 260 | 0.264 | 0.271 | 0.786 | 10.227 |
| 2 | 407 | 0.414 | 0.409 | 0.760 | 4.497 |
| 3 | 102 | 0.104 | 0.099 | 0.721 | 22.383 |
| 4 | 71 | 0.072 | 0.071 | 0.751 | 38.740 |
| 5 | 143 | 0.145 | 0.150 | 0.779 | 20.714 |
| **HAQ Group** |  |  |  |  |  |
| 1 | 112 | 0.114 | 0.124 | 0.810 | 33.063 |
| 2 | 332 | 0.338 | 0.325 | 0.740 | 5.589 |
| 3 | 291 | 0.296 | 0.297 | 0.709 | 5.789 |
| 4 | 191 | 0.194 | 0.189 | 0.747 | 12.227 |
| 5 | 57 | 0.058 | 0.064 | 0.812 | 70.499 |

**^a^** Estimated group probabilities should be close to the proportion of individuals classified in the group (proportion based on the assignments for the maximum posterior probability). **^b^** Average Posterior Probabilities (should be at least 0.7). **^c^** Odds of Correct Classification (should be ≥5.0). For 4 and 5 groups, Figure S3 contains the color legend regarding each trajectory.

**Table S7**. Univariate Multinomial Logistic Regression models for the association of baseline characteristics and physical function (HAQ) trajectories.

|  | Physical Function Trajectories (HAQ) | | |  |
| --- | --- | --- | --- | --- |
|  | **Consistently low disability**  **RRR (95% CI)** | **Slightly worsening moderate disability**  **RRR (95% CI)** | **Consistently high disability**  **RRR (95% CI)** | **p** |
| Sex |  |  |  | <0.001 |
| Male (ref) | - | - | - |  |
| Female | - | 2.70 (1.98, 3.67) | 5.34 (3.38, 8.44) |  |
| Age Class |  |  |  | <0.001 |
| <55 years old (ref) | - | - | - |  |
| 55-64 years old | - | 1.23 (0.82, 1.84) | 1.98 (0.98, 4.00) |  |
| 65-74 years old | - | 1.76 (1.18, 2.64) | 4.98 (2.57, 9.63) |  |
| ³75 years old | - | 2.99 (1.80, 4.97) | 14.81 (7.24, 30.33) |  |
| Region (NUTSII) |  |  |  | 0.036 |
| North (ref) | - | - | - |  |
| Centre | - | 1.00 (0.66, 1.50) | 0.76 (0.47, 1.25) |  |
| Lisbon | - | 0.60 (0.38, 0.93) | 0.64 (0.31, 0.92) |  |
| Alentejo | - | 1.15 (0.58, 2.29) | 1.18 (0.54, 2.61) |  |
| Algarve | - | 0.53 (0.17, 1.63) | 1.18 (0.39, 3.55) |  |
| Islands | - | 1.19 (0.79, 1.79) | 0.69 (0.41, 1.15) |  |
| Marital status |  |  |  | 0.002 |
| With partner | - | 0.89 (0.66, 1.22) | 0.54 (0.37, 0.78) |  |
| Educational Level |  |  |  | <0.001 |
| <4 years (ref) | - | - | - |  |
| 4-9 years | - | 0.43 (0.28, 0.67) | 0.17 (0.11, 0.27) |  |
| ³10 years | - | 0.22 (0.13, 0.38) | 0.03 (0.01, 0.08) |  |
| BMI (kg/m^2^) |  |  |  | <0.001 |
| Underweight/Normal weight (ref) | - | - | - |  |
| Overweight | - | 1.07 (0.71, 1.60) | 0.87 (0.52, 1.46) |  |
| Obese | - | 2.16 (1.42, 3.28) | 1.93 (1.15, 3.26) |  |
| Smoking habits |  |  |  | <0.001 |
| Never (ref) | - | - | - |  |
| In the past | - | 0.40 (0.28, 0.57) | 0.24 (0.14, 0.41) |  |
| Daily/Occasionally | - | 0.51 (0.30, 0.87) | 0.40 (0.19, 0.82) |  |
| Alcohol consumption |  |  |  | <0.001 |
| Never (ref) | - | - | - |  |
| Occasionally/Daily | - | 0.56 (0.42, 0.75) | 0.40 (0.28, 0.57) |  |
| Regular exercise (yes) |  | 0.51 (0.36, 0.71) | 0.27 (0.16, 0.44) | <0.001 |
| Clinical severity  (inverted KOOS/HOOS)  (0 low -100 high severity) | - | 1.06 (1.05, 1.07) | 1.10 (1.09, 1.12) | <0.001 |
| Unmanageable pain levels (yes) | - | 2.44 (1.76, 3.37) | 3.85 (2.43, 6.09) | <0.001 |
| Multimorbidity | - | 2.18 (1.58, 2.97) | 9.07 (5.01, 16.41) | <0.001 |
| RRR: Relative risk ratio; HAQ: Health Assessment Questionnaire; p – Wald test p-value. | | | |  |

**Table S8**. Univariate Multinomial Logistic Regression models for the association of HKOA patients baseline characteristics and HRQoL (EQ-5D) trajectories.

|  | HRQoL Trajectories (EQ-5D) | | |  |
| --- | --- | --- | --- | --- |
|  | **Consistently high**  **HRQoL**  **(Reference)** | **Consistently moderate**  **HRQoL**  **RRR (95% CI)** | **Consistently low**  **HRQoL**  **RRR (95% CI)** | **p** |
| Sex |  |  |  | <0.001 |
| Male (ref) | - | - | - |  |
| Female | - | 2.08 (1.44, 2.98) | 4.47 (2.92, 6.83) |  |
| Age Class |  |  |  | <0.001 |
| <55 years old (ref) | - | - | - |  |
| 55-64 years old | - | 1.24 (0.77, 2.01) | 1.65 (0.93, 2.91) |  |
| 65-74 years old | - | 1.49 (0.93, 2.39) | 2.59 (1.49, 4.50) |  |
| ³75 years old | - | 2.99 (1.56, 5.75) | 7.94 (3.93, 16.05) |  |
| Region (NUTSII) |  |  |  | 0.921 |
| North (ref) | - | - | - |  |
| Centre | - | 1.25 (0.76, 2.07) | 0.99 (0.58, 1.68) |  |
| Lisbon | - | 0.91 (0.54, 1.55) | 0.67 (0.38, 1.19) |  |
| Alentejo | - | 1.22 (0.54, 1.54) | 0.95 (0.39, 2.27) |  |
| Algarve | - | 0.80 (0.23, 2.72) | 0.83 (0.23, 2.98) |  |
| Islands | - | 1.14 (0.69, 1.87) | 0.93 (0.55, 1.58) |  |
| Marital status |  |  |  | <0.001 |
| With partner | - | 0.52 (0.35, 0.79) | 0.43 (0.28, 0.66) |  |
| Educational Level |  |  |  | <0.001 |
| <4 years (ref) | - | - | - |  |
| 4-9 years | - | 0.64 (0.38, 1.13) | 0.24 (0.14, 0.42) |  |
| ³10 years | - | 0.33 (0.17, 0.63) | 0.05 (0.02, 0.10) |  |
| BMI (kg/m^2^) |  |  |  | <0.001 |
| Underweight/Normal weight (ref) | - | - | - |  |
| Overweight | - | 0.76 (0.47, 1.22) | 0.90 (0.52, 1.57) |  |
| Obese | - | 1.27 (0.75, 2.12) | 2.65 (1.49, 4.72) |  |
| Smoking habits |  |  |  | <0.001 |
| Never (ref) | - | - | - |  |
| In the past | - | 0.47 (0.32, 0.71) | 0.32 (0.20, 0.51) |  |
| Daily/Occasionally | - | 1.56 (0.71, 3.41) | 0.97 (0.41, 2.29) |  |
| Alcohol consumption |  |  |  | <0.001 |
| Never (ref) | - | - | - |  |
| Occasionally/Daily | - | 0.49 (0.34, 0.70) | 0.40 (0.27, 0.59) |  |
| Regular exercise (yes) |  | 0.56 (0.39, 0.82) | 0.24 (0.15, 0.39) | <0.001 |
| Clinical severity  (inverted KOOS/HOOS)  (0 low -100 high severity) | - | 1.04 (1.03, 1.06) | 1.10 (1.08, 1.11) | <0.001 |
| Unmanageable pain levels (yes) | - | 3.00 (2.06, 4.37) | 5.33 (3.44, 8.28) | <0.001 |
| Multimorbidity | - | 1.58 (1.09, 2.28) | 6.17 (3.80, 10.01) | <0.001 |
| RRR – Relative risk ratio; HRQoL: Health Related Quality of Life; p – Wald test p-value. | | | |  |
